# Screen Time Is More than Just the Screen: Indirect Media Exposure Dominates Infants' Digital Environments

**DOI:** 10.1111/infa.70074

**Published:** 2026-02-07

**Authors:** Sarah C. Kucker, Julie M. Schneider

**Affiliations:** ^1^ Southern Methodist University Dallas Texas USA; ^2^ University of California Los Angeles Los Angeles California USA

**Keywords:** digital media, home media ecology, screen time

## Abstract

Digital media has become ubiquitous for families, yet little is known about how infants and toddlers are exposed to it across different contexts and demographics. Most existing research focuses narrowly on total screen time, often neglecting critical factors such as media type, user, timing, and background exposure. The current study sought to address this gap by asking 252 socioeconomically diverse caregivers of children 8–25 months about their child's media exposure, including how much, what kinds, when, by whom, and in what form digital media is used in the home. Consistent with prior work, infants averaged over 2 hours/day of direct media use but were exposed to even higher rates of indirect background TV (over 4.6 hours/day). Such background exposure represents 66%–75% of children's total TV and handheld device exposure and is significantly more common on weekends. However, there is substantial variability in children's media environments, with older infants being exposed to greater amounts and more diverse forms of media. Notably, children from lower‐SES households experience more background media and caregiver phone use, which may introduce both visual and auditory distractions during key developmental periods. This study highlights that the most prominent source of media exposure early in life is not through children's personal use of devices, but rather indirect exposure through background media use, which varies widely across families. To truly capture the landscape of young children's technology exposure and the impact on development, we must consider the broader media environment, not just the screen in front of the child.

## 1 Introduction

In the last decade, digital media has become a ubiquitous part of life for young children and their families. Current estimates show that over 97% of households with children have a television and 98% own a mobile device (Mann et al. 2025). By the time they are 2‐year‐olds, children use these devices themselves an average of 2 or more hours/day (Kucker et al. 2024) and are exposed to media nearly 4–5 hours/day indirectly as background noise (Dore and Dynia 2021; Lapierre et al. 2012; Nichols 2022; Ribner et al. 2021). Digital media is thus a critical part of the child's daily ecology (Barr 2019).

Despite the widespread presence of digital media in young children's lives, detailed research on the context of their exposure remains limited. The majority of work on children's digital media use has focused on total amount of screen time, often measured simply as a parental report of children's own use as low, medium, or high (e.g., Takahashi et al. 2023) or through recording devices such as LENA which records the number of minutes of electronic sounds picked up by the device (Sundqvist et al. 2021; Ramirez et al. 2021; Brushe et al. 2024). While useful, these measures offer only a coarse snapshot, overlooking critical details such as what types of media children are exposed to (e.g., TV, games, e‐books), when during the day exposure occurs, why media is being used, and who is engaging with it—the child or those around them. These factors are known to matter when it comes to children's development. For instance, although educational media can support language growth (Jing et al. 2023), lower‐quality video viewing has been linked to poorer vocabulary skills compared to more interactive media (Kucker et al. 2024). Similarly, higher caregiver media use is associated with more behavior problems in children (Kildare and Middlemiss 2017) and greater technoference—media‐based disruptions to caregiver‐child interactions—which negatively impacts both language development and behavior (Corkin et al. 2021; McDaniel and Radesky 2018). Yet much of the existing literature overlooks contextual factors, such as types of media, when media is used, and who is using it, especially when highlighting variability across families (Coyne et al. 2017; Barr and Kirkorian 2023; Lauricella et al. 2015).

In addition to types of screen time and media content, the sensory characteristics of digital media—such as visual overstimulation or background auditory input—can introduce environmental distractions that compete with children's attention and learning. These sensory features can affect children differently depending on developmental stage and context. Indeed, prior work has found that non‐digital background noise and visual distractions effect multiple elements of development, with white noise hampering word learning (Riley and McGregor 2012), and more visual distractions lowering attention and learning in a classroom (Fisher et al. 2014). In media, fast‐paced visuals, quick scene changes, and background noise can disrupt attention and working memory in infants and toddlers (Christakis et al. 2004; Lillard et al. 2015; Courage et al. 2010). Similarly, auditory distractions from background television or caregiver phone use have been shown to reduce the quantity and quality of caregiver‐child interactions and child language input (Kirkorian et al. 2009; Pempek et al. 2014; McDaniel and Radesky 2018). These forms of sensory distraction may have differential effects on cognitive, linguistic, and emotional development—effects that may not be captured by traditional screen time metrics alone. Therefore, a nuanced understanding of how media is presented, not just how much, is essential for identifying pathways of influence on child development.

The lack of such details in our understanding of digital media use is particularly critical because not all families use the same forms of digital media, at the same rates, or times. In fact, there is substantial variability in media use across children of different ages (Kucker et al. 2024; Mann et al. 2025), racial backgrounds, and socioeconomic (SES) groups (Yang‐Huang et al. 2018). As children get older, the amount of time they spend using digital media rises rapidly; by age 8, 68% of children own their own tablet and use media directly 3.5 h/day (Mann et al. 2025). As they reach school age, children also get more autonomous in their media use leading to more time using video games/apps and the use of media for formal schooling (Mann et al. 2025). However, far less work has documented these developmental changes in children under 3 years of age—a period of extraordinary brain growth and heightened sensitivity to environmental input. Rates of digital media use further vary based on household demographics. For instance, lower SES households report higher rates of digital media use (Dore and Dynia 2021) and significantly higher use of background TV than higher SES households—an effect that is apparent even in infancy (Lapierre et al. 2012). There have also been reported differences in the types of media used—4–5‐year‐old children from lower SES neighborhoods are significantly more likely than children living in higher SES neighborhoods to be video game users and less likely to be computer users (Carson et al. 2010). These differences in media exposure can have cascading effects on development: higher media use has been linked to lower vocabulary skills (Madigan et al. 2020), and this relationship is especially pronounced among children from lower SES backgrounds (Dore and Dynia 2021). Moreover, SES appears to moderate the impact of digital media on behavioral outcomes such as curiosity, with higher levels of media use more strongly associated with lower curiosity among children from under‐resourced families than among their higher‐SES peers (Shah et al. 2021), suggesting that the developmental consequences of media use are not uniform across children. Together, these patterns highlight an urgent need for developmentally sensitive, equity‐focused research that captures when, how, and for whom digital media exposure shapes early learning and brain development.

Taken together, understanding the household media ecology and broader contexts in which media is used—including background media use and media use by caregivers—can give a more accurate and comprehensive picture of children's media exposure, and potential risks. This idea is instantiated by what the Dynamic Relational Ecological Approach to Media Effects Research model calls the family media ecology (DREAMER; Barr et al. 2024). In this model, the broader context and qualities in which media is used by both the child and the caregiver, as well as broader structural differences such as SES, all play a role in directing children's media exposure and subsequent outcomes. Without a broader picture of what kinds, when, and by who media is used, especially at a very young age and across SES, we are limited in understanding the possible cascading effects of digital media use on development.

### 1.1 Current Study

Despite the rapid increase in media use and its' established impacts on developmental milestones, minimal work has measured variability in media use across both indirect and direct exposure, especially when considering age and SES differences during the first two years. Using a large sample of young infants, we quantify the landscape of children's current media exposure, including how much, what kinds, when, by whom, and in what form, children are exposed to digital media. Such a comprehensive picture aims to give us a better understanding of the digital media ecology and thus adjust recommendations and research accordingly.

## 2 Methods

### 2.1 Participants

A total of 330 caregivers of children 8–25‐month completed a series of questionnaires via Prolific between November 2024 and February 2025. Of those, 78 participants were dropped for failing validity checks as noted below, a rate consistent with prior work using online samples (Chmielewski and Kucker 2020). This left a final sample of 252 caregivers (*n* = 180 female) of children 8–25‐month (*M* = 16.35 months, SD = 3.11; *n* = 120 female). This sample size is sufficient to detect a small‐medium effect based on G*Power 3.1.9.7 for a linear regression with an alpha of 0.05, power of 0.95, and up to 4 predictors. Caregivers were primarily White and non‐Hispanic but varied in SES. Income‐to‐needs ratio (ITN) served as a proxy for SES and was computed by dividing each participant's reported household income by the federal poverty threshold corresponding to their household size, consistent with standard practice (e.g., Duncan and Magnuson 2012). Poverty thresholds were based on the U.S. Department of Health and Human Services federal poverty guidelines for the year of data collection. An ITN ratio of 1.0 indicates income at the poverty line, values below 1.0 reflect income below the poverty threshold, and values closer to 0 indicate more severe economic disadvantage. Higher ITN ratios reflect greater economic security. Age in months was not correlated with ITN (*R* = −0.06, *p* = 0.35). See Table 1 for demographic information. Caregivers who completed the survey were paid $5. The study was conducted in accordance with the ethical standards of the American Psychological Association and was approved by the IRB at Southern Methodist University.

**TABLE 1:** Demographic information of the final sample.

		Primary caregiver	Child
Race	White	*N* = 192	*N* = 196
	Black	*N* = 41	*N* = 50
	Asian	*N* = 8	*N* = 13
	American Indian	*N* = 2	*N* = 2
	Hawaiian	*N* = 0	*N* = 2
	Multiracial	*N* = 7	*N* = 28
	Not listed	*N* = 2	*N* = 4
Ethnicity	Hispanic	*N* = 20	*N* = 29
Gender	Female	180	120
	Male	72	130
	Not listed/NR	0	2
Age		31.50 years (20–79); 7.13	16.35 months (8.03–25.12); 3.11
Income‐to‐needs ratio		3.12 (0.13–7.69); 1.69	

*Note:* 23 caregivers reporting being a single parent and thus no information on a secondary caregiver was given. Mean (range); Standard Deviation.

In order to ensure data were valid, we first screened participants using Prolific's built‐in qualification criteria and then cleaned the data thoroughly according to recommendations (Chmielewski and Kucker 2020). Only participants that listed their youngest child was born in 2023, noted English as their primary language, and had an approval rate of 95%–100% were able to view the study. After completion, responses were checked for (1) consistency in reporting their child's date of birth which was asked three times across different sections of the survey (*n* = 29 dropped), (2) eligibility with a child within the date range and primarily monolingual (*n* = 27 dropped), or (3) odd responses (no variation in responses, illogical answers to open ended questions, inconsistent name of child; *n* = 22 dropped).

### 2.2 Measures/Procedure

Each caregiver completed a series of questionnaires with demographic questions about themselves and their child (including age and demographic information about SES), their household routines, and a set of questions based on the validated Media Assessment Questionnaire 2.4 (Barr et al. 2024) that tapped the types of media activities children engage in, as well as children's exposure to both direct and indirect media. Caregiver use of media was also assessed. An overview of survey questions are available via OSF: https://osf.io/fnr2p/overview?view_only=4de47b1a909b4ac686d740b7144fc156.

#### 2.2.1 Assessing How Much Media, What Kinds, and When It Is Used

To assess the amount and types of media used, caregivers provided the average number of minutes their child engages in various digital and non‐digital activities on an average weekday and weekend. Activities reported included: watching videos (i.e., including movies, series, TV shows, home videos or video clips) on any device (i.e., YouTube, TV, cellphone, or computer), reading or looking at print books, and playing without digital media or books. They were also asked, on average, how many minutes during a weekday or weekend the child is exposed to background TV (i.e., indirect media use).

#### 2.2.2 Assessing Who Is Using Media

To assess who is using each form of media, caregivers reported which forms of media, and which devices (handheld device, TV, computer, gaming console, or other), were used by their child, others around their children, or other individuals when not around the child. This was reported as an overall percentage across the sample.

#### 2.2.3 Assessing Indirect Media Use in Detail

We then asked further follow‐up questions about children's indirect exposure to background television as it represents one of the most common forms of media exposure for children at this age (Dore and Dynia 2021; Mann et al. 2025). To capture indirect media exposure, caregivers were asked to report about the overall number of minutes per weekday and weekend day they spend in various activities with their child on a daily basis (e.g., playing, eating, chores), and then asked to report the proportion of time that the TV was on in the background during those activities. To probe the extent to which TV represented a possible distraction or interference for interaction, we measured how often it might present a visual distraction (with no audio being heard) or auditory distraction (no visual TV seen) only. These questions asked the percent of time the TV was visible but on mute/low audio and how often the TV was not visible but could be heard. Caregiver's were also asked to report their smartphone use and access by reporting information on their screen time use as measured by their phone, and asking the amount of time it was present during interactions, the time it could be heard but was out of reach, and the time it could be seen but was on silent.

## 3 Results

### 3.1 How Much, What Kinds, and When Is Digital Media Used?

We first sought to examine the prevalence of direct and indirect media use in comparison to other, non‐media‐related activities, and whether these patterns differ from a weekday to a weekend. Caregivers reported which types of media were used by their child (videos/TV, games, video chat, e‐books) as well as time exposed (hour/minutes per day) to background TV as a measure of indirect media exposure. Reported time‐use measures showed coherent patterns, with substantial portions of the day devoted to play and background media, alongside more limited time spent in direct screen use and book reading (see Table 2 for average minutes per day), suggesting that caregivers were able to meaningfully distinguish among the activity categories.

**TABLE 2:** Average minutes children spend in activities across days.

	Weekday	Weekend	Overall average
Direct media use	140.08 (121.03)	144.98 (118.99)	141.48 (115.46)
Book reading	99.01 (106.85)	100.73 (111.19)	99.50 (106.57)
Playing alone	332.32 (186.65)	338.00 (189.14)	333.94 (183.05)
Indirect media use	281.05 (189.16)	332.62 (190.69)	295.79 (184.66)

*Note:* Mean (SD).

Using a linear mixed effects model with overall average duration as the outcome, type as the fixed effect, and a random intercept for subject, we found significant differences in the duration of use across types of activities. We used pairwise comparisons with a Bonferroni correction to clarify which activities children were more or less likely to engage in. Children play alone significantly more than any other activity (background TV [*B* = 38.2, *p* = 0.006]; book reading [*B* = 234.4, *p* < 0.0001]; watching TV [*B* = 192.5, *p* < 0.0001]). However, the prevalence of indirect media exposure closely followed, with children experiencing significantly more exposure to background TV as compared to watching TV themselves (*B* = 154.3, *p* < 0.0001) and reading/looking at print books (*B* = 196.3, *p* < 0.0001). Direct media use, such as watching TV, was also more frequent at this age then reading/looking at print books (*B* = 42.0, *p* = 0.002). We ran the same analysis for duration of each activity on weekdays and weekends, independently. On weekdays, the same pattern of results emerged (see Supporting Information S1: Tables S1 and S2). Interestingly, on weekends, the frequency of indirect media use compared to playing alone did not significantly differ (*B* = 5.38, *p* = 0.66; see Supporting Information S1: Tables S3 and S4). Thus, while playing alone is the most frequent activity children engage in overall, on weekends, the frequency of indirect media use increases substantially and does not differ from frequency of playing alone.

To investigate whether the frequency of activities varies as a function of age or SES, we ran two independent linear mixed effects models. Each model included *overall* average duration as the outcome (mean duration of all media activities), with an interaction term between type and age, or type and SES, as the fixed effect, and a random intercept for subject. There were no significant differences in frequency of activity as a function of age (Supporting Information S1: Table S5) or SES (Supporting Information S1: Table S6).

### 3.2 By Whom Are Different Types of Digital Media Used?

Given the prevalence of digital media in children's environments, we further explored what types of digital media use occur most often in households, and whether these types of digital media are most often used directly by children, by others in the child's presence, or by others when the child is not present. Binomial responses were recorded (i.e. device uses by a given individual = 1; device not used by a given individual = 0), and the percent of caregivers who reported digital media use by type and person was computed (see Table 3 for percentages). Caregivers reported that children are directly exposed to digital media most often in the form of TV (*M* = 37.6%, SD = 48.5%), but indirectly in the form of handheld device use by others in their presence (*M* = 75.3%, SD = 43.2%). Gaming consoles, while present in the child's environment, are primarily used by other family members when the child is not present (*M* = 55.0%, SD = 49.9%). Overall, caregivers reported their child is significantly more likely to be exposed to digital media by others in their environment than through their personal use of devices (*t*(251) = −16.02, *p* < 0.0001; see Table 3 and Supporting Information S1). More specifically, caregivers stated their child is exposed to approximately 295.79 min of indirect digital media use (SD = 115.46 min) compared to 141.48 min of direct digital media use (SD = 115.46 min; *t*(251) = −15.58, *p* < 0.0001). The systematic variation in reported exposure across device types and social contexts—consistent with how these technologies are typically used and aligning with other reports (Mann et al. 2025)—suggests that caregivers were able to meaningfully distinguish among survey options.

**TABLE 3:** Child exposure to technology by type and person.

	Child uses themselves	Child is around others who use it	Is used by family/others, but not around the child
Handheld devices	24.7% (43.2%)	75.3% (43.2%)	19.9% (40.0%)
Stationary TV	37.6% (48.5%)	66.0% (47.5%)	18.4% (38.8%)
Computer	3.6% (18.7%)	50.8% (50.1%)	51.6% (50.1%)
Gaming console	4.2% (20.0%)	47.9% (50.1%)	55.0% (49.9%)
Other	29.8% (45.8%)	46.9% (50.0%)	37.1% (48.4%)

*Note:* Mean (SD). Percent of individuals reporting each type/person is reported.

We next evaluated whether direct or indirect media use on any of these devices varied as a function of child age or SES. A linear regression with an interaction term of device type used by the child and age in days predicting frequency indicated that the frequency of handheld device significantly increases as children in this age group develop (*b* = 0.001, *p* = 0.004). Stationary TV (*b* = 0.001, *p* = 0.07) and other devices (*b* = 0.001, *p* = 0.07) moderately increased with age as well. A linear regression examining this same interaction based on use by others around the child did not significantly differ as a function of age. When investigating the moderating role of SES, we find that, for devices used by the child, higher SES is associated with lower use of stationary TVs (*b* = −0.04, *p* = 0.04). Use of devices by others around the child did not vary as a function of SES.

### 3.3 When and in What Modality Is Indirect Media Present During Daily Activities?

**FIGURE 1:**
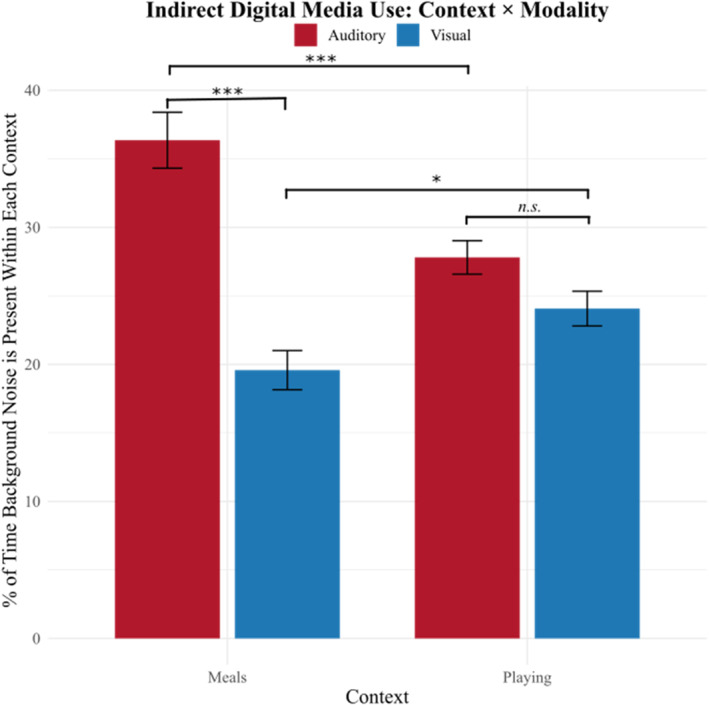
Exposure to media indirectly across contexts and modalities. Black bars represent standard error. n.s. = not significant. *p < 0.05, ***p < 0.001.

To more granularly explore when during the day indirect exposure to digital media is most common, and how it is experienced, we asked caregivers to report the percent of time digital media is present in the background during specific activities, and whether it was present as either an (1) auditory distraction (can be heard, but not seen) or (2) visual distraction (can be seen, but not heard). Importantly, this activity‐based and modality‐specific framing allowed caregivers to report on concrete, familiar experiences rather than abstract judgments. We find that, when children are playing, indirect media is visually present in children's environments 22.71% of the time (SD = 18.11%) and during mealtimes, indirect media is present 19.50% of the time (SD = 19.78%). During playtime, indirect media is auditorily present in children's environments 27.53% of the time (SD = 17.96%) and 35.91% during mealtime (SD = 28.42%; see Figure 1). A linear regression with an interaction term between modality (visual/auditory) and context (meals/play) predicting frequency revealed a significant interaction (*b* = 13.04, *p* < 0.0001), as well as main effects of modality (*b* = −16.78, *p* < 0.0001) and context (*b* = −8.55, *p* < 0.0001). Follow‐up comparisons revealed that during meals, indirect media exposure was significantly more likely to be auditory than visual (*b* = 16.78, *p* < 0.0001), whereas during play, auditory and visual exposure occurred at comparable rates (*b* = 3.74, *p* = 0.084). Auditory indirect media was more prevalent during meals than play (*b* = 8.55, *p* = 0.0001), while visual indirect media was more prevalent during play than meals (*b* = −4.48, *p* = 0.038).

**FIGURE 2:**
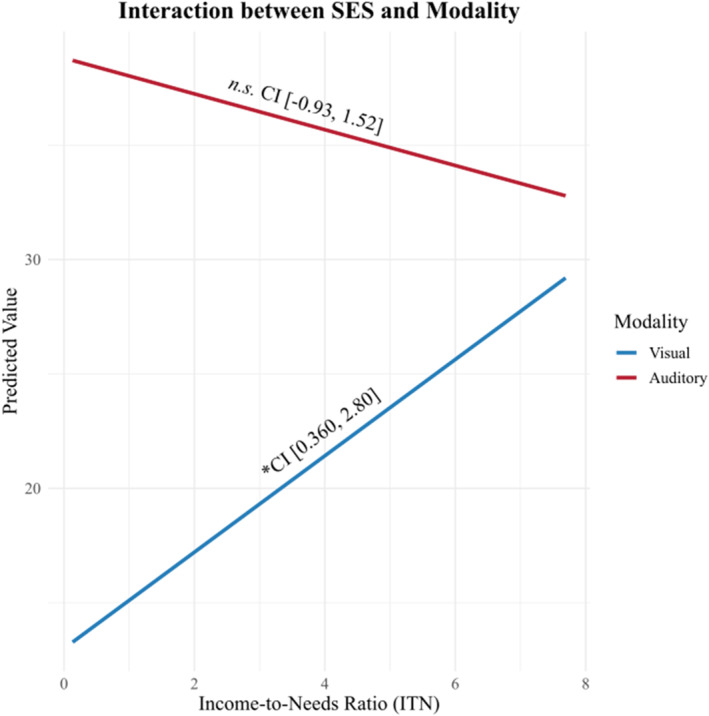
Differences in digital media distraction across modality and SES. n.s. = not significant. * = significant.

We also sought to determine whether these patterns of context and modality varied as a function of child age (in days) or SES. When including child age as a third interaction term (with modality and context), we find no significant changes in the pattern of digital media use across age (see Supporting Information S1: Table S7). When including SES as a third interaction term, we find a significant interaction between modality and SES (*b* = 2.89, *p* = 0.02), as well as similar results to the above analysis, including an interaction between context and modality (*b* = 23.04, *p* < 0.001), and main effects of modality (*b* = −25.80, *p* < 0.0001) and context (*b* = −15.29, *p* < 0.001; for full output see Supporting Information S1: Table S8). To probe the significant modality and SES interaction, we examined the simple slopes of SES within each modality using the emtrends() function of the emmeans R package (Lenth 2024). Results indicated that SES was positively associated with the frequency of visual distractions, such that children from higher SES households experienced increasing levels of visual background media exposure (*b* = 1.582, SE = 0.622, 95% CI [0.360, 2.80]; Figure 2). In contrast, SES was not significantly related to the frequency of auditory distractions (*b* = 0.295, SE = 0.622, 95% CI [−0.927, 1.52]). These findings suggest that SES (as indexed by ITN) differentially impacts digital media use depending on the modality of input, with stronger effects observed for visual distractions.

Given the rise of cell phone use, which may further introduce indirect media use into children's lives, we also asked caregivers to report how often they actively use their cell phone when playing with their child, how often it is present visually versus aurally, and the degree to which their cell phone distracts them when interacting with their child. When asked to report their average weekly screen time from their phones, caregivers reported using their phone approximately 489.72 min. (SD = 380.31), stating they actively used it 38.67% of the time when playing with their child (SD = 23.30%). Caregiver screen time only increased as a function of child age (*R* = 0.13, *p* = 0.04), but did not differ on the basis of SES (*R* = −0.02, *p* = 0.70). Caregivers further reported that, even if they weren't actively using their phone, it may still present a distraction when playing with their child by being visually present 56.53% of the time (SD = 26.84%) and aurally present 67.35% of the time (SD = 30.37%).

## 4 Discussion

The current study provides a comprehensive understanding of the digital media ecology in the daily lives of infants and toddlers. By assessing both direct and indirect forms of media exposure across a large, socioeconomically diverse sample of caregivers, our findings reveal that the most prominent form of media exposure during infancy is not personal device use, but rather background or indirect exposure to digital media through TV and device use by others. This indirect exposure—particularly background TV—was not only more frequent than direct media use but also highly variable across families and contexts. Importantly, we found that exposure to media is not uniformly distributed throughout the day or across activities; distractions from digital media are most often present in the auditory modality during meals, but visually during play, suggesting meaningful variation in how media may interfere with key learning moments and caregiver–child interactions.

Our findings build upon and extend prior work, expanding our understanding of the details of children's media exposure. While previous studies have focused primarily on minutes of screen time, we show that a focus on coarse measures of screen time fail to capture the complex ways in which digital media is present in children's environments. Caregivers in our sample universally reported having smartphones within reach during playtime, with many acknowledging that their device was a distraction even when not in active use. The presence of such indirect digital media use—whether through caregiver devices or background TV—represents a subtle but consistent intrusion into early learning contexts, potentially reducing opportunities for language‐rich and responsive interactions (Barr 2019; Dore and Dynia 2021).

Of particular interest is the differential pattern of media exposure across SES, operationalized here as an income‐to‐needs ratio. Importantly, we do not interpret this measure as suggesting that income per se directly causes differences in parenting or children's media environments; rather, income‐to‐needs is widely used as a proxy for a broader set of structural conditions that shape daily life, including access to material resources, time and bandwidth, housing density and household “chaos,” childcare availability, and caregiver stress and wellbeing. These factors are known to influence both the quality of the home learning environment and the quantity/quality of caregiver–child interaction—pathways that are central to early language and self‐regulation development (Pace et al. 2017; Rowe 2012; Rowe and Weisleder 2020; Sarsour et al. 2011; Schneider et al. 2024). Within this framework, higher TV use among lower SES households in the current study may reflect constraints that make digital media use more likely (e.g., crowded or multi‐use living spaces, fewer alternative childcare/enrichment options, or routines organized around caregiver workload), rather than differences in parental values or intentions. In addition, structural factors such as nonstandard or unpredictable work schedules—which are more common in lower‐wage occupations—can disrupt routines and increase caregiver fatigue/stress, with downstream impacts on parent–child interactions and the organization of the home environment (Grzywacz et al. 2011; Ugarte and Hastings 2024; Wang 2023).

By contrast, the tendency for higher SES caregivers to report more visual distractions (e.g., “TV on mute”) than lower SES caregivers may reflect different forms of multitasking and household organization (e.g., media used as ambient visual content while preserving a quieter auditory environment), suggesting distinct pathways through which media ecology intersects with learning opportunities (Barr et al. 2024). In line with the DREAMER framework, these patterns underscore that who is using media, what type of media is present, when it is present (routines/activities), and how it is experienced (auditory vs. visual disruption) may be more informative than global screen‐time estimates—particularly when examining equity‐relevant differences in exposure.

Although developmental guidelines recommend minimizing screen exposure before age two, our findings indicate that even within this narrow window of infancy and toddlerhood, digital media exposure *increases* with age. We observed that direct media use rises as children get older, yet this increase does not come at the expense of indirect exposure. Rather than replacing background media, direct screen time appears to be layered on top of existing exposure, resulting in higher overall media exposure across the first two years of life. This cumulative exposure—occurring during a sensitive period for language, attention, and social development—raises important concerns given prior evidence linking early media exposure to delays in vocabulary growth and self‐regulation (Madigan et al. 2020; Dore and Dynia 2021).

We also found that both objective (device‐recorded screen time from caregivers' phones) and subjective (caregiver self‐report of cell phone use during interactions) measures of caregiver phone use increased as children aged, a pattern that may reflect several underlying factors. The inclusion of device‐based screen time estimates is a notable strength of the current study, as self‐reports of technology use are known to be vulnerable to recall error and social desirability bias. The parallel age‐related increases observed across both measurement approaches suggest that this trend reflects genuine changes in caregiver behavior rather than reporting artifacts. Several mechanisms may underlie this pattern. One possibility is that as infants become more mobile and capable of independent play, caregivers may naturally shift their attention toward their devices during downtime. For example, research has shown that caregivers increase their use of technology for the purpose of communication as their children develop (Rudi et al. 2015). Alternatively, caregivers of older infants may feel more comfortable acknowledging their media use, either because they perceive it as less detrimental or because they have developed more confidence in their parenting practices. Or, perhaps as children get older, there are increases in behavior problems, which is known to increase parent stress, which in turn increases media use (McDaniel and Radesky 2020). Regardless of the reason, this trend suggests that media exposure in early childhood is not static—but instead intensifies with age, both in terms of child‐directed use and ambient digital distractions introduced by caregivers.

While the present study offers a rich snapshot of the early digital media environment, there are also several limitations. First, although our sample was socioeconomically diverse, it was disproportionately White and composed of monolingual families, which may limit generalizability to other racial, ethnic, and linguistic populations. Second, this study is cross‐sectional and cannot speak to the long‐term developmental impacts of indirect media exposure. Longitudinal work is needed to examine how early exposure to background media—especially in different modalities and routines—affects later cognitive, linguistic, and social outcomes (McDaniel and Radesky 2020; Takahashi et al. 2023). Finally, while we examined variability in how and when media is present, we did not assess media content, which may play a critical role in moderating its effects on development, and did not assess motivations for media use, which prior work has found might moderate the effect of children's media use on their development (Suh et al. 2024; Kucker et al. 2024). Future studies should consider not only the quantity and context of exposure but also the quality and purpose of media use in the lives of young children.

The current study raises critical implications for research methodology and public health guidance. Our results support a shift away from unitary screen time recommendations (e.g., < 1 h/day) and towards more nuanced, ecologically valid metrics that account for the indirect and variable nature of media exposure. This is particularly critical given prior work showing that background noise, even from non‐media sources, can impact children's development (Erickson and Newman 2017); here we confirm that media is a prevalent source of such distraction. For researchers, this means designing studies that capture not just child device use, but also the broader media landscape. For clinicians and policy makers, this means offering caregivers actionable guidance on minimizing disruptive background media, rather than only focusing on limiting active screen time use.

## Author Contributions

**Sarah C. Kucker:** conceptualization, investigation, funding acquisition, writing – original draft, methodology, writing – review and editing, data curation. **Julie M. Schneider:** conceptualization, investigation, writing – original draft, methodology, writing – review and editing, data curation, data analysis, visualization.

## Funding

This study was supported by a National Institute of Child Health and Human Development (R15HD101841) grant to SCK.

## Ethics Statement

The study was conducted in accordance with the ethical standards of the American Psychological Association and with approval from the Southern Methodist University IRB.

## Conflicts of Interest

The authors declare no conflicts of interest.

## Supporting information


Supporting Information S1

## Data Availability

Survey tools, data, and analytical scripts are available via OSF: https://osf.io/fnr2p/overview?view_only=4de47b1a909b4ac686d740b7144fc156.
