# Supplementary material for: Screen Time Is More than Just the Screen: Indirect Media Exposure Dominates Infants' Digital Environments
Source: Infancy. 2026 Feb 7;31(1):e70074. doi: 10.1111/infa.70074 (PMC12882697; doi:10.1111/infa.70074)
Supplement: Supplementary file 1 — Supporting Information S1 [file INFA-31-0-s001.docx]

**Supplemental Materials**

*Supplementary Table 1.*

Duration of child activities on weekdays only.

| **Activity** | **Estimate** | **Std. Error** | ***t*-value** | ***p*-value** |
| --- | --- | --- | --- | --- |
| Intercept | 281.05 | 9.79 | 28.70 | < 0.0001 |
| Book Reading | -182.04 | 11.70 | -15.57 | < 0.0001 |
| Playing Alone | 51.27 | 11.70 | 4.38 | < 0.0001 |
| Indirect Media Use | -140.97 | 11.70 | -12.05 | < 0.0001 |

These results are based on a linear mixed effects model with weekday average duration as the outcome, type as the fixed effect, and a random intercept for subject. The intercept is duration of direct media use.

*Supplementary Table 2.*

Pairwise comparisons between frequency of activities on weekdays only.

| **Contrast** | **Estimate** | **Std. Error** | ***t*-value** | ***p*-value** |
| --- | --- | --- | --- | --- |
| Indirect Media – Book Reading | 182.00 | 11.70 | 15.57 | < 0.0001 |
| Indirect Media – Playing Alone | -51.30 | 11.70 | -4.38 | 0.0001 |
| Indirect Media – Direct Media | 141.00 | 11.70 | 12.05 | < 0.0001 |
| Book Reading – Playing Alone | -233.30 | 11.70 | -19.95 | < 0.0001 |
| Book Reading – Direct Media | -41.10 | 11.70 | -3.51 | 0.003 |
| Playing Alone – Direct Media | 192.20 | 11.70 | 16.44 | < 0.0001 |

*Note*: All comparisons were adjusted using a Bonferroni correction.

*Supplementary Table 3.*

Duration of child activities on weekends only.

| **Activity** | **Estimate** | **Std. Error** | ***t*-value** | ***p*-value** |
| --- | --- | --- | --- | --- |
| Intercept | 332.62 | 9.89 | 33.62 | < 0.0001 |
| Book Reading | -231.89 | 12.14 | -19.10 | < 0.0001 |
| Playing Alone | 5.38 | 12.14 | 0.44 | 0.66 |
| Indirect Media Use | -187.64 | 12.14 | -15.46 | < 0.0001 |

These results are based on a linear mixed effects model with weekend average duration as the outcome, type as the fixed effect, and a random intercept for subject. The intercept is duration of direct media use.

*Supplementary Table 4.*

Pairwise comparisons between frequency of activities on weekends only.

| **Contrast** | **Estimate** | **Std. Error** | ***t*-value** | ***p*-value** |
| --- | --- | --- | --- | --- |
| Indirect Media – Book Reading | 231.88 | 12.10 | 19.10 | < 0.0001 |
| Indirect Media – Playing Alone | -5.38 | 12.10 | -0.44 | 1 |
| Indirect Media – Direct Media | 187.64 | 12.10 | 15.46 | < 0.0001 |
| Book Reading – Playing Alone | -237.26 | 12.10 | -19.54 | < 0.0001 |
| Book Reading – Direct Media | -44.25 | 12.10 | -3.65 | 0.0017 |
| Playing Alone – Direct Media | 193.02 | 12.10 | 15.90 | < 0.0001 |

*Note*: All comparisons were adjusted using a Bonferroni correction.

*Supplementary Table 5.*

Linear regression model examining variability in frequency of different activities across child age (in days).

|  | **Estimate** | **Std. Error** | ***t*-value** | ***p*-value** |
| --- | --- | --- | --- | --- |
| (Intercept) | 244.80 | 51.34 | 4.77 | 0.00*** |
| Book Reading | -152.40 | 61.62 | -2.47 | 0.01* |
| Playing Alone | 43.09 | 61.62 | 0.70 | 0.48 |
| Direct Media Use | -157.30 | 61.62 | -2.55 | 0.01* |
| Age (in Days) | 0.10 | 0.10 | 1.01 | 0.31 |
| Book Reading x Age | -0.09 | 0.12 | -0.73 | 0.47 |
| Playing Alone x Age | -0.01 | 0.12 | -0.08 | 0.94 |
| Direct Media Use x Age | 0.01 | 0.12 | 0.05 | 0.96 |

*Supplementary Table 6.*

Linear regression model examining variability in frequency of different activities across child SES (measured as ITN).

|  | **Estimate** | **Std. Error** | ***t*-value** | ***p*-value** |
| --- | --- | --- | --- | --- |
| (Intercept) | 299.70 | 20.18 | 14.86 | < 2e-16*** |
| Book Reading | -223.31 | 24.20 | -9.23 | < 2e-16 *** |
| Playing Alone | 11.53 | 24.20 | 0.48 | 0.63 |
| Direct Media Use | -166.44 | 24.20 | -6.88 | 0.00*** |
| SES (ITN) | -1.25 | 5.69 | -0.22 | 0.83 |
| Book Reading x SES | 8.66 | 6.82 | 1.27 | 0.21 |
| Playing Alone x SES | 8.53 | 6.82 | 1.25 | 0.21 |
| Direct Media Use x SES | 3.89 | 6.82 | 0.57 | 0.57 |

*Supplementary Table 7.*

Linear regression model examining variability in modality and context of digital media use across child age (in days).

|  | **Estimate** | **Std. Error** | ***t*-value** | ***p*-value** |
| --- | --- | --- | --- | --- |
| (Intercept) | 27.63 | 8.01 | 3.45 | 0.00*** |
| Context | 4.72 | 11.32 | 0.42 | 0.68 |
| Modality | 1.96 | 11.32 | 0.17 | 0.86 |
| Age | 0.02 | 0.02 | 1.11 | 0.27 |
| Context * Modality | -9.97 | 16.01 | -0.62 | 0.53 |
| Context * Age | -0.03 | 0.02 | -1.19 | 0.23 |
| Modality * Age | -0.04 | 0.02 | -1.69 | 0.09 |
| Context * Modality * Age | 0.05 | 0.03 | 1.46 | 0.14 |

*Supplementary Table 8.*

Linear regression model examining variability in modality and context of digital media use across SES (measured as ITN).

|  | **Estimate** | **Std. Error** | ***t*-value** | ***p*-value** |
| --- | --- | --- | --- | --- |
| (Intercept) | 38.81 | 3.14 | 12.35 | < 2e-16*** |
| Context | -15.29 | 4.44 | -3.44 | 0.00*** |
| Modality | -25.80 | 4.44 | -5.81 | 0.00*** |
| SES | -0.78 | 0.88 | -0.89 | 0.37 |
| Context * Modality | 23.04 | 6.29 | 3.67 | 0.00*** |
| Context * SES | 2.15 | 1.24 | 1.73 | 0.08 |
| Modality * SES | 2.89 | 1.24 | 2.32 | 0.02* |
| Context * Modality * SES | -3.20 | 1.76 | -1.82 | 0.07 |
